# Transthoracic Resection versus Non-Transthoracic Resection for Gastroesophageal Junction Cancer: A Meta-Analysis

**DOI:** 10.1371/journal.pone.0037698

**Published:** 2012-06-04

**Authors:** Kun Yang, Hai-Ning Chen, Xin-Zu Chen, Qing-Chun Lu, Lin Pan, Jie Liu, Bin Dai, Bo Zhang, Zhi-Xin Chen, Jia-Ping Chen, Jian-Kun Hu

**Affiliations:** 1 Department of Gastrointestinal Surgery, West China Hospital, Sichuan University, Chengdu, Sichuan Province, People’s Republic of China; 2 West China School of Medicine, Sichuan University, Chengdu, Sichuan Province, People’s Republic of China; University of York, United Kingdom

## Abstract

**Background:**

The aim of this meta-analysis is to evaluate the impact of transthoracic resection on long-term survival of patients with GEJ cancer and to compare the postoperative morbidity and mortality of patients undergoing transthoracic resection with those of patients who were not undergoing transthoracic resection.

**Method:**

Searches of electronic databases identifying studies from Medline, Cochrane Library trials register, and WHO Trial Registration *etc* were performed. Outcome measures were survival, postoperative morbidity and mortality, and operation related events.

**Results:**

Twelve studies (including 5 RCTs and 7 non-RCTs) comprising 1105 patients were included in this meta-analysis, with 591 patients assigned treatment with transthoracic resection. Transthoracic resection did not increase the 5-y overall survival rate for RCTs and non-RCTs (HR = 1.01, 95% CI 0.80- 1.29 and HR = 0.89, 95% CI 0.70- 1.14, respectively). Stratified by the Siewert classification, our result showed no obvious differences were observed between the group with transthoracic resection and group without transthoracic resection (P>0.05). The postoperative morbidity (RR = 0.69, 95% CI 0.48- 1.00 and OR = 0.55, 95% CI 0.25- 1.22) and mortality (RD =  −0.03, 95% CI −0.06- 0.00 and RD = 0.00, 95% CI −0.05- 0.05) of RCTs and non-RCTs did not suggest any significant differences between the two groups. Hospital stay was long with thransthoracic resection (WMD =  −5.80, 95% CI −10.38- −1.23) but did not seem to differ in number of harvested lymph nodes, operation time, blood loss, numbers of patients needing transfusion, and reoperation rate. The results of sensitivity analyses were similar to the primary analyses.

**Conclusions:**

There were no significant differences of survival rate and postoperative morbidity and mortality between transthoracic resection group and non-transthoracic resection group. Both surgical approaches are acceptable, and that one offers no clear advantage over the other. However, the results should be interpreted cautiously since the qualities of included studies were suboptimal.

## Introduction

Gastroesophageal junction (GEJ) cancer has been gradually considered as an entity separate from both esophageal cancer and gastric cancer [1]. Although a decline in incidence of gastric carcinoma, there has been a tendency of proximal migration of carcinoma in Western countries [2]–[4]. A kind of classification proposed by Siewert & Stein, which includes three types, was widely accepted for GEJ cancer [5]. According to the classification, type 1 is defined as tumors whose centers are located 1 to 5 cm above the gastroesophageal junction (distal esophageal adenocarcinoma); type 2, adenocarcinoma with its epicenter located between 1 cm proximal and 2 cm distal of the GEJ, is defined as a true cardia carcinoma; and the center of the type 3 tumor lies 2 to 5 cm distal to the GEJ (subcardial gastric carcinoma) [5].

Surgery is the mainstay treatment although the prognosis is poor. Controversies, especially on operation route, still exist. The debate on the question whether transthoracic (TT) resection or non- transthoracic resection is better for GEJ cancer remains continuing. Transthoracic resection was advocated with intent to prolong the survival, because mediastinal lymph nodes could be observed and dissected under the direct vision and a safe surgical margin is easy to obtain in the operation process [6]–[9]. Non-transthoracic resection, such as transhiatal resection or transabdominal resection, was recommended since it could decrease the respiratory complications related to transthoracic resection and the damage caused by the anastomotic leakage [9]–[14]. In addition, positive metastasis of mediastinal lymph nodes indicated a poor prognosis even though the dissection was complete [9], [15]. Although some randomized controlled trials (RCTs) had not showed the superiority of transthoracic resection to non-transthoracic resection for Siewert type 1 and 2 GEJ cancers, transthoracic resection did show better trend for survival [12], [14], [16]. Nevertheless, Sasako M *et al* demonstrated the opposite results for type 2 and 3 GEJ cancers [15]. And Goldfaden *et al* also claimed transhiatal esophagectomy performed a better survival advantage for type 1 patients in spite of no statistical difference [17]. For safety, several studies have shown a lower incidence of complications with non-transthoracic resection [13]–[15]. However, some authors argued that the transthoracic resection technique do not increase the morbidity and mortality [11], [12], [18].

So there is still uncertainty in aspects of survival and safety between transthoracic resection and non-transthoracic resection for GEJ cancers. The aim of this meta-analysis is to evaluate the impact of transthoracic resection on long-term survival of GEJ cancer and to compare the postoperative morbidity and mortality of patients undergoing transthoracic resection with those of patients who were not undergoing transthoracic resection.

## Methods

In order to guarantee the quality, this meta-analysis was conducted in line with recommendations from the Cochrane Collaboration and the Quality of Reporting Meta-Analyses (QUOROM) statement [19].

**Figure 1:**
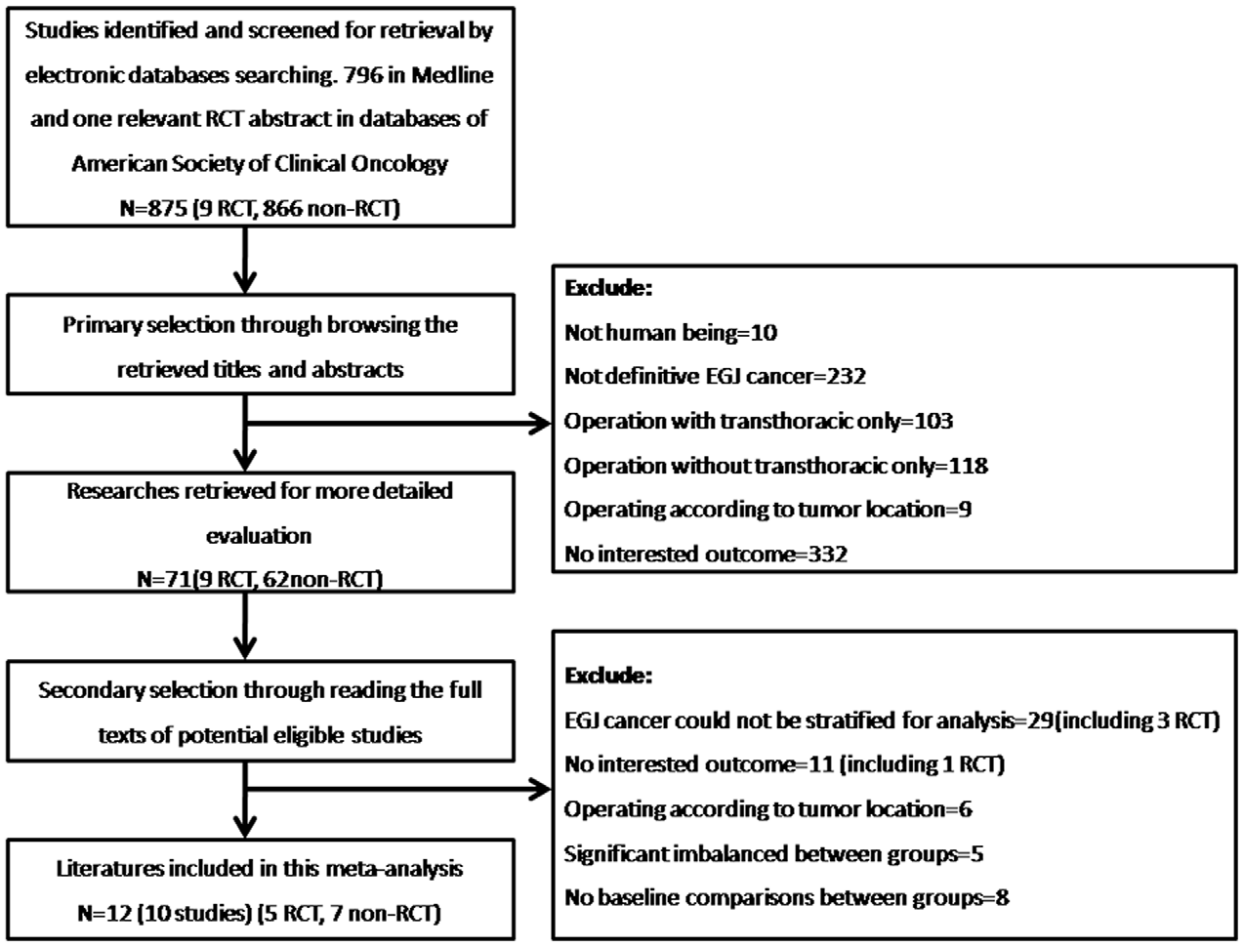
Flow chart for selection of studies. The flowchart of selecting procedure and the exclusive reason of studies are summarized.

**Table 1:** The characteristics of included trials.

Study	Methods	Patients		Siewert Classification	Stage	Residual tumor	Chemotherapy		Matching^a^	Primary endopoints	Quality
		TT	Non-TT				TT	Non-TT			
Chu KM, 1997 [12]	RCT	19	20	1	Early to advanced	NR	NR	NR	1,2,4,8,9	Median Survival/safety,NS	-
Hulscher JB, 2002 [14]/Omloo JMT,2007 [16]	RCT	114	106	1, 2	0, I-IV	R0, R1, R2	0	0	1,2,4,5,6,7,8,9,10,11	OS/DFS, NS	-
JCOG 9502, 2006 [15]	RCT	85	82	2, 3	I-IV	R0, R1	2	0	1,2,3,4,5,6,7,8,9,10,11	OS, NS but favorNon-TT	-
Goldfaden D, 1986 [17]	Retrospective	43	29	1, 2	I-IV	NR	NR	NR	1,2,4,6,7,8,9	Survival/safety, FavorNon-TT	4
Okamura T, 1989 [31]	Retrospective	39	95	1, 2, 3	NR	R0, R1, R2	NR	NR	1,2,3,6,7,10	Survival/safety, FavorTT on survival	3
Rizzetto C, 2008 [32]	Retrospective	40	18	1, 2	0, I-III	R0	40	18	2,4,6,7,8,9,11	OS, Favor TT	4
Stark SP, 1996 [33]	Retrospective	16	32	1, 2	NR	R0, R1	8	10	1,2,4,5,7,8,9,10,11	Survival/safety,NS but favor TT	7
Johansson J, 2004 [34]	Retrospective	27	22	1, 2	T3N1M0	NR	NR	NR	2,3,4,6,7,8,9	OS, Favor TT	5
Moon MR, 1992 [35]	Retrospective	24	63	1	I-IV	NR	NR	NR	1,2,4,5,6,7,8,9	Survival/safety, NS	6
Zheng B, 2010 [36]	Retrospective	284	47	2	I-IV	R0, R1	NR	NR	1,2,3,4,6,7,8,9,11	Survival/safety, NS	7

**Abbreviations:** RCT: Randomized controlled trial; TT: Transthoracic resection; Non-TT: Non-Transthoracic resection; NR: Not reported; NS: No significant; OS: Overall survival; DFS: Disease-free survival.

a 1, age; 2, gender; 3, tumor size; 4, tumor location; 5, comorbidity; 6, depth of tumor invasion; 7, lymph node metastasis; 8, distant metastasis; 9, stage; 10, differentiation; 11, curative degree.

**Table 2:** The quality of included randomized trials.

Study	Truly random	Concealed allocation	Baselinefeatures	Eligibilitycriteria	Blinding assessment	Loss tofollow-up	Intensionto treat	Studyquality
Chu KM, 1997 [12]	Unclear	Unclear	Yes	No	Unclear	No	Yes^a^	Poor
JCOG 9502, 2006 [15]	Yes	Unclear	Yes	Yes	No	Yes	Yes	Fair
Hulscher JB, 2002 [14]/Omloo JMT, 2007 [16]	Yes	Unclear	Yes	Yes	Unclear	Yes^b^	Yes^c^	Fair

a All patients underwent the planned procedure.

b All patients have completed the follow up.

c The whole patients were analyzed by an intention to treat analysis. However, per protocol analysis was applied when the patients were stratified by the location of tumors.

**Figure 2:**
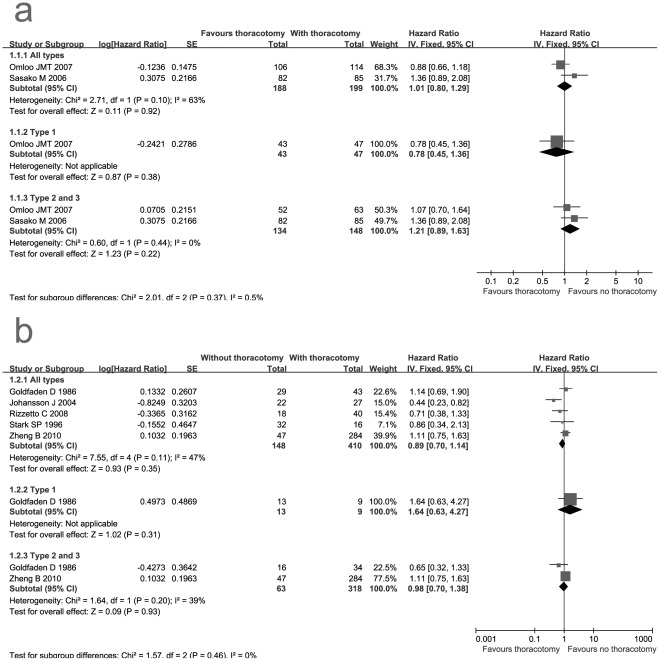
Forest plot of 5-year overall survival rates for RCTs and non-RCTs. a: RCTs; b: non-RCTs. The 95% confidence interval (CI) for the hazard ratio for each study is represented by a horizontal line and the point estimate is represented by a square. The size of the square corresponds to the weight of the study in the meta-analysis. The 95% CI for pooled estimates is represented by a diamond. Data for a fixed-effects model are shown as there was no statistical heterogeneity. df = degrees of freedom; I^2^ = percentage of the total variation across studies due to heterogeneity; IV = Inverse Variance; SE = standard error; Z = test of overall treatment effect.

**Table 3:** The Meta-results of effectiveness and safety of transthoracic resection for patients with EGJ cancer.

			Number of studies	WithoutTT n/N	WithTT n/N	HR/RR/OR/RD[95% CI]	P-value for effect size	I square	P-value for heterogeneity	Effect model
**RCT**										
		All Siewert types	2	188^a^	199^a^	1.01 [0.80, 1.29]^b^	0.92	63%	0.10	Fixed
	**Survival**	Type 1	1	43^a^	47^a^	0.78 [0.45, 1.36]^b^	0.38	–	Not applicable	Fixed
		Type 2 and 3	2	134^a^	148^a^	1.21 [0.89, 1.63]^b^	0.22	0%	0.44	Fixed
	**Safety**	Overall morbidity	1	28/82	42/85	0.69 [0.48, 1.00]^c^	0.05	–	Not applicable	Fixed
		Hospital or postoperative 30 days mortality	3	2/208	8/218	−0.03 [−0.06, 0.00]^d^	0.09	0%	0.80	Fixed
**Non-RCT**										
		All Siewert types	5	148^a^	410^a^	0.89 [0.70, 1.14]^b^	0.35	47%	0.11	Fixed
	**Survival**	Type 1	1	13^a^	9^a^	1.64 [0.63, 4.27]^b^	0.31	–	Not applicable	Fixed
		Type 2 and 3	2	63^a^	318^a^	0.98 [0.70, 1.38]^b^	0.93	39%	0.20	Fixed
	**Safety**	Overall morbidity	4	78/234	133/390	0.55 [0.25, 1.22]^e^	0.14	70%	0.02	Random
		Hospital or postoperative 30 days mortality	5	20/210	19/406	0.00 [−0.05, 0.05]^d^	0.87	8%	0.36	Fixed

**Abbreviations:** TT: Transthoracic resection; HR: Hazard ratio; RR: Risk ratio; OR: Odds ratios; RD: Risk difference.

a the summed number of patients in each group.

b HR.

c RR.

d RD.

e OR.

**Figure 3:**
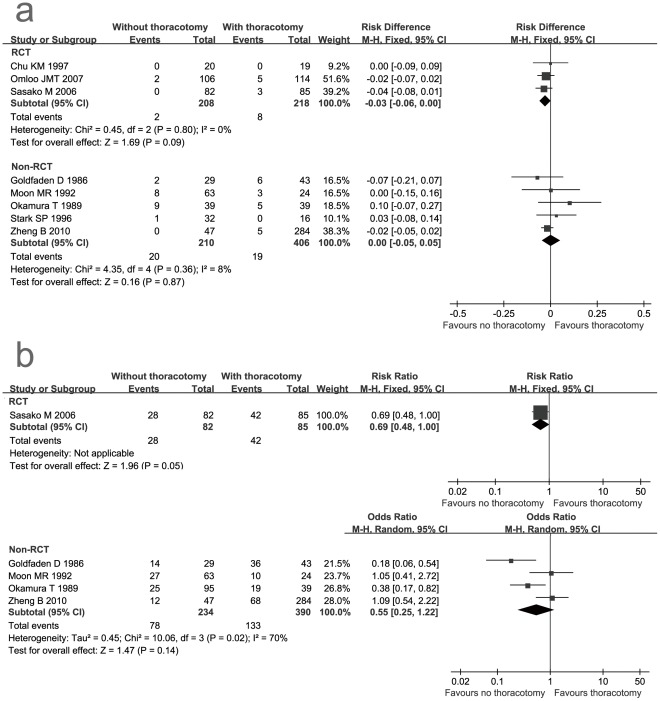
Forest plot of mortality and morbidity. a: mortality; b: morbidity. The 95% confidence interval (CI) for the risk difference, risk ratio or odds ratio for each study is represented by a horizontal line and the point estimate is represented by a square. The size of the square corresponds to the weight of the study in the meta-analysis. The 95% CI for pooled estimates is represented by a diamond. Data for a fixed-effects model are shown as there was no statistical heterogeneity. Data for a random-effects model are shown as there was statistical heterogeneity. df = degrees of freedom; MH = Mantel-Haenszel test; I^2^ = percentage of the total variation across studies due to heterogeneity; Z = test of overall treatment effect.

**Table 4:** Pooled estimates of common specific postoperative complications.

	Number of studies	WithoutTT n/N	With TTn/N	OR/WMD/RD [95% CI]	P-value foreffect size	I square	P-value for heterogeneity	Effect model
Anastomotic leakage	7	53/454	47/605	0.93 [0.59, 1.45]^c^	0.74	0	0.60	Fixed
Chylous leakage	1	2/106	11/114	0.18 [0.04, 0.83]^c^	0.03	–	Not applicable	Fixed
Wound infection	4	10/202	18/460	−0.02 [−0.06, 0.02]^d^	0.36	19%	0.29	Fixed
Length of ICU stay (day)	1	106^a^	114^a^	−4.00 [−6.70, −1.30]^b^	0.004	–	Not applicable	Fixed
Cardiac complications	5	33/265	89/484	0.69 [0.43, 1.10]^c^	0.12	0	0.89	Fixed
Pulmonary complications	8	75/474	129/624	0.62 [0.27, 1.43]^c^	0.26	70%	0.001	Random
Hoarseness	4	27/218	28/200	0.02 [−0.10, 0.14]^d^	0.78	70%	0.02	Random
Mechanical ventilation required	2	7/102	16/104	0.41 [0.16, 1.03]^c^	0.06	0	0.49	Fixed
Duration of intubation	2	138^a^	130^a^	1.26 [−3.43, 5.94]^b^	0.60	81%	0.02	Random

**Abbreviations:** TT: Transthoracic resection; OR: Odds ratios; WMD: Weighted mean difference; RD: Risk difference.

a the summed number of patients in each group.

b WMD.

c OR.

d RD.

**Table 5:** Outcomes of Meta-analysis of operation related events.

	Number of studies	Without TT n/N	With TT n/N	OR/WMD/RD [95% CI]	P-value foreffect size	I square	P-value for heterogeneity	Effect model
Number of harvested lymph nodes	5	289^a^	526^a^	−5.05 [−13.07, 2.97]^b^	0.22	96%	P<0.00001	Random
Operation time (min)	4	181^a^	404^a^	−23.92 [−77.23, 29.40]^b^	0.38	99%	P<0.00001	Random
Blood loss (ml)	4	178^a^	431^a^	−41.27 [−139.76, 57.21]^b^	0.41	94%	P<0.00001	Random
Number of patients needing transfusion	2	39/114	43/101	0.98 [0.23, 4.20]^c^	0.97	75%	0.04	Random
Length of hospital stay (day)	5	224^a^	487^a^	−5.80 [−10.38, −1.23]^b^	0.01	91%	P<0.00001	Random
Reoperation	2	1/67	4/303	−0.01 [−0.06, 0.04]^d^	0.71	61%	0.11	Fixed

**Abbreviations:** TT: Transthoracic resection; OR: Odds ratios; WMD: Weighted mean difference; RD: Risk difference.

a the summed number of patients in each group.

b WMD.

c OR.

d RD.

**Table 6:** Summary of data of trials without extractable information for Meta-analyses.

	WithoutTT N	WithTT N	WithoutTT Median	WithTT Median	P-value
**Operation time (min)**					
Omloo JMT, 2007^16^	106	114	210	360	<0.001
**Blood loss (ml)**					
Omloo JMT, 2007^16^	106	114	1000	1900	<0.001
**Length of hospital stay (day)**					
Stark SP, 1996^80^	32	16	15	14	NS
Moon MR, 1992^82^	63	24	18	18	NS

**Abbreviations:** TT: Transthoracic resection; NS: No significant.

**Table 7:** Results of sensitivity analysis for overall survival rates and safety.

		Number of studies	Without TT n/N	With TT n/N	HR/RR/OR/RD [95% CI]	P-value for effect size	I square		P-value for heterogeneity	Effect model	
**Combining RCT and non-RCT**											
Hazard ratio for 5-y overall survival		7	336^a^	609^a^	0.93 [0.73, 1.19]^b^	0.57	44%		0.09	Random	
Overall morbidity		5	106/316	175/475	0.55 [0.31, 0.99]^c^	0.04	61%		0.04	Random	
Hospital or postoperative 30 days mortality		8	22/418	27/624	−0.01 [−0.04, 0.02]^d^	0.44	0%		0.70	Fixed	
**High quality studies**											
	Hazard ratio for 5-y overall survival	2	188^a^	199^a^	1.01 [0.80, 1.29]^b^	0.92	63%		0.10	Fixed	
**RCT**	Overall morbidity	1	28/82	42/85	0.69 [0.48, 1.00]^e^	0.05	–		Not applicable	Fixed	
	Hospital or postoperative 30 days mortality	2	2/188	8/199	−0.03 [−0.06, 0.00]^d^	0.08	0%		0.75	Fixed	
	Hazard ratio for 5-y overall survival	2	79^a^	300^a^	1.07 [0.75, 1.52]^b^	0.72	0%		0.61	Fixed	
**Non-RCT**	Overall morbidity	2	39/110	78/308	1.07 [0.61, 1.90]^c^	0.80	0%		0.95	Fixed	
	Hospital or postoperative 30 days mortality	3	9/142	8/324	0.01 [−0.05, 0.04]^d^	0.84	0%		0.60	Fixed	
**Studies with direct statistic method**											
**RCT**	Hazard ratio for 5-y overall survival	2	188^a^	199^a^	1.01 [0.80, 1.29]^b^	0.92	63%		0.10	Fixed	
**Non-RCT**	Hazard ratio for 5-y overall survival	1	18^a^	40^a^	0.71 [0.38, 1.33]^b^	0.29	–		Not applicable	Fixed	
**Studies matched stage**											
	Hazard ratio for 5-y overall survival	2	188^a^	199^a^	1.01 [0.80, 1.29]^b^	0.92	63%		0.10	Fixed	
**RCT**	Overall morbidity	1	28/82	42/85	0.69 [0.48, 1.00]^e^	0.05	–		Not applicable	Fixed	
	Hospital or postoperative 30 days mortality	3	2/208	8/218	−0.03 [−0.06, 0.00]^d^	0.09	0%		0.80	Fixed	
	Hazard ratio for 5-y overall survival	5	148^a^	410^a^	0.89 [0.70, 1.14]^b^	0.35	47%		0.11	Fixed	
**Non-RCT**	Overall morbidity	3	53/139	114/351	0.63 [0.22, 1.80]^c^	0.38	75%		0.02	Random	
	Hospital or postoperative 30 days mortality	4	11/171	14/367	−0.02 [−0.07, 0.03]^d^	0.45	0%		0.70	Fixed	
**Final survival result (15 year) of JCOG9502 was considered**											
**RCT**	Hazard ratio for 5-y overall survival	2	188^a^	199^a^	1.08 [0.71, 1.64]^b^	0.73	69%		0.07	Random	
**RCT**	Hazard ratio for type 2 and 3 cancers	2	134^a^	148^a^	1.22 [0.93, 1.62]^b^	0.41	0%		0.16	Fixed	

**Abbreviations:** TT: Transthoracic resection; HR: Hazard ratio; RR: Risk ratio; OR: Odds ratios; RD: Risk difference.

a the summed number of patients in each group.

b HR.

c OR.

d RD.

e RR.

### Search Strategy and Study Selection

We searched electronic databases of PubMed. The further websites and conference proceedings were searched, including Cochrane Central Register of Controlled Trials, National Cancer Institute, European Organization for Research and Treatment of Cancer, Southwest Oncology Group, ClinicalTrials.gov, American Society of Clinical Oncology, and European Society of medical Oncology. Moreover, the reference lists from relevant articles were screened for eligibility. The eligible unpublished grey papers which were prevented from publication because of conflict of interests were considered to be included also, if known to Prof. Chen ZX and Prof. Chen JP.

The search strategy of Medline was as follows and was also applied to other databases: [((“Gastroesophageal Junction”[Mesh] AND “Carcinoma”[Mesh]) OR (“Stomach Neoplasms”[Mesh] AND “Carcinoma”[Mesh]) OR (“Cardia”[Mesh] AND “Carcinoma”[Mesh]) OR (“Carcinoma”[Mesh] AND “Esophageal Neoplasms”[Mesh]) ) OR ((((((((gastroesophageal junction) OR gastroesophageal junction) OR ogastroesophageal junction) OR distal esophageal) OR lower third esophageal) OR cardia) OR subcardial) OR siewert)] AND (((((transhiatal [Title/Abstract]) OR transabdominal[Title/Abstract]) OR transthoracic[Title/Abstract]) OR thoracoabdominal[Title/Abstract]) OR abdominothoracic[Title/Abstract]). The electronic search was up to November, 2011 with no limitations on publication date and language.

### Inclusion and Exclusion Criteria

Any kinds of articles including RCTs, controlled clinical trials, Cohort studies, case-control studies, and case series which compared the effectiveness or safety of transthoracic resection to those of non-transthoracic resection were eligible.

The inclusion criteria were as follows: Tumor should locate at distal esophagus, cardia or subcardia as Siewert classification. The patients had to have histological proven adenocarcinoma as reported in the texts. The patients treated with chemotherapy, immunotherapy *etc* perioperatively were included. The patients received thoracotomy or non-thoracotomy. There was no limitation of age, gender, and race. Curative & palliative operations were included. One or more outcome measures should be extracted. And the exclusion criteria contained: Recruited patients with carcinoma at other location of stomach or esophagus, such as gastric antrum or middle/upper third of esophagus or EGJ cancer could not be stratied for analysis were excluded. Patients with metachronous or synchronous double cancer were excluded. Patients with other significant comorbidities, such as benign diseases or other kinds of tumors (lymphoma etc) were excluded. Patients whose operations were performed according to the location of tumors were excluded. Trials possessing uncertainty or important inequality of characteristics on baselines between groups were excluded.

### Selection, Assessment, and Data Extraction

In order to select studies for further assessment, two independent reviewers screened the title, abstract section, and keywords of every record retrieved. Full articles were assessed if the given information suggested that the study conformed to our criteria described above.

Any disagreements in quality assessment and data collection were discussed and solved by a third reviewer (Hu JK and Zhang B) as the referee.

Data was extracted independently by two reviewers. Details of study sample (number of each arm, study population characteristics, and matching items), interventions (the detail of operation, as well as details of other treatment, such as adjuvant chemotherapy etc.) and outcomes (5-year overall survival rate, postoperative mortality and morbidity, and operation related events) were extracted. Additionally, first author, the year of study, study design, the number and reason of withdrawals, dropouts and their characteristics were extracted.

Seven items relevant to the quality appraisal were used for assessing [20]: 1) whether the method of allocation was truly random; 2) whether there was proper concealment of allocation; 3) whether there was equality between two groups at baseline in terms of prognostic features; 4) whether the eligibility criteria were described; 5) whether blindness of the outcome assessors was performed; 6) whether loss to follow-up in each treatment arm was demonstrated, and 7) whether intention-to-treat analysis was considered. Studies with seven or six ‘yes’ was required for a trial to be rated as high quality, while five or four ‘yes’ for fair quality and three or fewer ‘yes’ for low quality [20].

The qualities of non-randomized studies were assessed by using the Newcastle-Ottawa Scale (NOS) with some modifications to match the needs of this study [21]. The quality of the studies was evaluated by examining patient selection methods, comparability of the study groups, and assessment of outcomes. Studies achieving six or more scores were considered to be of relative high quality.

### Outcomes of Interest and Definitions

The primary outcome measures were 5-year overall survival rate, overall hospital or postoperative 30 day mortality, and overall morbidity rate, while the secondary outcome measure were specific operation related events, including number of harvested lymph nodes, operation time, intra-operative blood loss, number of patients needing transfusion, length of hospital stay, and reoperation rate. One or more outcome measures were required in the included trials, or they were excluded.

### Statistical Analysis

For dichotomous data, relative risks (RR) for RCTs and odds ratios (OR) for non-RCTs which were weighted estimates of treatment effect across trials were calculated. If the RR or OR could not be estimated because of a low incidence event in either group, the risk difference (RD) was calculated instead [22]. Continuous data was calculated as weighted mean differences (WMD) with 95% confidence intervals. The analyses were conducted using RevMan 5.0 provided by the Cochrane Collaboration [23]. The P value<0.05 was considered as statistically significant. The overall survival rates were calculated as hazard ratios (HR). If only survival curves were reported, the overall 5-year survival rates were extracted and converted from the figures as accurately as possible [24]. When the trials had reported medians and ranges instead of means and standard deviations, the means and standard deviations (SD) were calculated according to Hozo SP *et al* [25]. If the data could not be extracted for meta-analysis, we presented the results in a descriptive and qualitative manner [26]. Heterogeneities of treatment effect between trials were tested using a Chi-squared statistic with significance being set at P<0.10, and I-square was used to estimate total variation across studies that was due to heterogeneity rather than chance (<25% was considered as low level heterogeneity, 25% to 50% as moderate level, and higher than 50% as high level) [27]. If heterogeneities existed, one of the following techniques was undertaken to attempt to explain: 1.Random effect model for meta-analyses was considered; 2. Sub-group analyses; 3. Sensitivity analyses were considered. The subgroup analyses were considered to be performed stratified by the Siewert classification. Some common specific postoperative complications were also analyzed in the subgoup analysis. Sensitivity analyses were performed according to the following aspects: refer to high quality trials only to avoid the misleading caused by poor quality studies [28], combine RCT and non-RCT studies, use studies with direct statistic method only, utilize studies matched stage only, or apply the final survival results of JCOG9502 trial instead of results of the first interim analysis. Tests for funnel plot asymmetry were planned to be used only when there are at least ten studies included in each meta-analysis [29].

## Results

### Study Selection

There were 875 studies identified in total using the predefined search strategy (including 9 RCTs and 866 non-RCTs). Checking the references of retrieved studies did not provide any further studies for evaluation. Subsequently, selection was performed according to the inclusion/exclusion criteria set out in the Methods section. Through browsing the retrieved titles and their abstracts, 804 articles were excluded at the primary selection step, followed by exclusion of a further 59 articles at the secondary selection step, which involved reading the full texts of potentially eligible studies. The flowchart of selecting procedure and the exclusive reason of studies are summarized in Figure 1.

Twelve studies (including 5 RCTs and 7 non-RCTs) meeting the inclusion criteria were chosen [12], [14]–[17], [30]–[36]. No grey papers were found. Two non-RCT studies with imbalanced baseline of comorbidity were included since postoperative morbidity and mortality were not their outcomes and cancer-related deaths were compared [32], [34]. The publication years of these studies ranged from 1986 to 2010. Because some publications reported on the same trial and the same patient groups differed only in analyzed parameters, only 9 studies were applicable [14]–[16], [30]. The characteristics and quality assessments of included trials were listed in Table 1 and 2.

A total of 1105 patients were available for analysis, with 591 patients treated with transthoracic resection (considered as control group in this meta-analysis).

### Survival

The meta-analysis of RCTs and non-RCTs showed there were no significant survival benefits for the group with transthoracic resection (HR = 1.01, P = 0.92 and HR = 0.89, P = 0.35 respectively). However, the pooled result of non-RCTs proned to favor the group with transthoracic resection (HR = 0.89, 95% CI: 0.70- 1.14), which could not be observed from the result of RCTs (Table 3, Figure 2). As the Siewert classification of GEJ cancer has a major effect on the choice of operation procedure [37], we performed the subgroup analysis stratified by the Siewert classification.

In the subgroup analysis, we also could find, for type 1 GEJ cancer, transthoracic resection could not facilitate survival compared with non-transthoracic resection from the results of RCTs and non-RCTs (Table 3, Figure 2). The HRs of 5-year overall survival rates for RCT and non-RCT were 0.78 and 1.64 respectively. Because type 2 cancers are considered more similar to type 3 cancers [37], we combined these two types to analyze. Our result showed no obvious differences were observed between the group with transthoracic resection and group without transthoracic resection for type 2 and 3 GEJ cancers (Table 3, Figure 2).

For those studies which were not included in the meta-analysis, we had listed their results as follows:

The JCOG9502 reported the final survival result (15 year) which showed the hazard ratio was 1.36 (95%CI: 0.94- 1.99) in favor of non-transthoracic resection in spite of insignificant difference (P = 0.95) [30].

One RCT reported the median survival times were 18 (transhiatal) and 13.5 (transthoracic) months respectively with no significant difference [12].

One non-RCT showed the 5-y survival rates were 12% and 16% for transhiatal and transthoracic groups respectively (P>0.05) [35].

### Safety

Both meta-analyses of RCTs and non-RCTs showed that transthoracic resection did not significantly influence 30 day mortality (RD = −0.03, 95% CI: −0.06- 0.00, P = 0.09 and RD = 0.00, 95% CI: −0.05- 0.05, P = 0.87 respectively) (Table 3, Figure 3). Transthoracic resection was associated with a higher overall postoperative morbidity than non-transthoracic resection from the results of RCTs and non-RCTs (RR = 0.69, 95% CI: 0.48- 1.00, P = 0.05 and OR = 0.55, 95% CI: 0.25- 1.22, P = 0.14 respectively), but these differences were not statistical significant (Table 3, Figure 3).

For those studies which were not included in the meta-analysis, the morbidity of one RCT did not show any significant differences between transthoracic and transhiatal group [12]. Another RCT and non-RCT reported there were no significances between transthoracic and transhiatal group on postoperative morbidities, except pulmonary complication [14], [33].

Furthermore, subgroup analyses were performed for some common specific postoperative complications. These analyses showed that the incidences of complications, such as anastomotic leakage, wound infection, cardiovascular complications, and hoarseness were not significantly different between patients treated with transthrocotomy and those without transthrocotomy (P>0.05). Even with respect to pulmonary complications, the incidences were not significant different between the two groups. Also, the numbers of patients requiring mechanical ventilation and durations of intubation postoperatively did not differ statistically between the two groups (P>0.05). Patients undergoing transthrocotomy seemed to experience more chylous leakage and longer ICU stay than patients without transthrocotomy (P<0.05) (Table 4).

### Operation Related Events

Hospital stay was long with thransthoracic resection (WMD = −5.80, 95% CI −10.38- −1.23) but did not seem to differ in number of harvested lymph nodes, operation time, blood loss, numbers of patients needing transfusion, and reoperation rate (Table 5). Data of other trials without extractable information for meta-analyses had been summarized in Table 6.

### Sensitivity Analysis

When high-quality studies, studies with direct statistic method, studies matched for stage or the final survival results of JCOG9502 considered were re-performed for meta-analyses in sensitivity analyses, the results were shown in Table 7. No changes of outcomes were observed in terms of the 5-y overall survival rate, postoperative morbidity and mortality, compared to the primary results. When RCT and non-RCT studies were combined, there was a significant increase in postoperative morbidity with thransthoracic resection (OR = 0.55, 95% CI: 0.31- 0.99, P = 0.04). This contrasts with the lack of evidence of difference in 5-year overall survival and 30 day mortality (Table 7).

## Discussion

The incidence of GEJ cancers has been increased [2]–[4]. Surgery is still considered as the potential curative treatment. Proximal margin length and lymph node involvement are independent prognostic factors for GEJ cancers [16], [38]. So, transthoracic resection permitting the safe margin and a more clearance of involved nodes is proposed to benefit the survival rate of patietns with GEJ cancer. However, the results did not suggest better survival sometimes [15], [17], [33]. Furthermore, non-transthoracic resection theoretically minimizes respiratory complications, decreases risk of anastomotic leakage, reduces the incidence of postoperative symptoms associated to gastroesophageal reflux, and avoids a painful incision of transthoracic resection [33]. Still, some authors reported that the transthoracic resection technique did not increase the morbidity and mortality [11], [12], [18]. So, there is still discrepancy on which one is the best approach.

With respect to the 5-year overall survival rate, our results failed to suggest transthoracic resection could bring more benefit to the patients with GEJ cancers, which was in accordance with the recent published results [39]. To avoid the bias caused by the Siewert classification, we performed the subgroup analysis. When stratified by the Siewert classification, transthoracic resection, which even though was proved to not increase the survival rates of patients with type 1 cancers, also showed the potential survival benefit from the results of RCTs (HR = 0.78, 95% CI: 0.45- 1.36). Because type 1 cancers tend to have more positive metastasis lymph nodes locating at the middle or upper mediastinum [15], [40], the potential more clearance of involved nodes of transthoracic resection for type 1 cancer patients strengthened the survival advantage [9], [15], [16], [41]. And for type 2 and 3 cancers, our results also showed no obvious differences were observed between the groups with transthoracic resection and without transthoracic resection for survival. However, the result of RCTs has showed possible survival advantage for the group without transthoracic resection (HR = 1.21, 95% CI: 0.89- 1.63). This may derive from the relatively skeptical quality and less scientific authority of non-RCTs, although we indeed carefully select some non-RCTs with good balanced baseline characteristics for meta-analysis. And also may because that type 2 or 3 tumors were more commonly presented as undifferentiated and less intestinal growth pattern, greater depth of serosa invasion, higher lymph node burden, more frequent advanced stages, and lower R0 resection rates [37], the effect of non-transthoracic resection was a little diminished from the result of non-RCTs. Furthermore, the power of outcomes was restricted by the limited number of included studies and the relative small sample size. Nonetheless, the overall survival effects were not impacted by transthoracic resection and the authors believed the results of survival presented in this meta-analysis could be referred to with caution, after all. In the sensitivity analysis, no significant differences could be detected in 5-year overall survival rates between non-transthoracic resection group and transthoracic resection group.

Regarding to the safety, the present meta-analysis showed transthoracic resection did not significantly influence mortality and morbidity. However, there was a marginal benefit of morbidity for non-transthoracic resection (RR = 0.69, 95% CI: 0.48- 1.00, P = 0.05 and OR = 0.55, 95% CI: 0.25- 1.22, P = 0.14 respectively). Although some authors reported there were no significant differences of pulmonary complications between transthoracic resection and non-transthoracic resection [11]–[13], [18], more pulmonary complications were observed following transthoracic resection [14], [15], [39], [42]. Furthermore, transthoracic resections can result in a transient deterioration of pulmonary function during one-lung ventilation in the left-lateral position compared with non-transthoracic resection approach, although this might be partly compensated for during the intervention [18]. However, our subgroup analysis stratified by specific postoperative complications demonstrated the incidences of pulmonary complcations were not significant different between the two groups. Also, the numbers of patients requiring mechanical ventilation and durations of intubation postoperatively did not differ statistically between the two groups. Nevertheless, the relative higher heterogeneity might compromise the validity of the results. And we should interpret cautiously. There seemed to be a higher incidence of chylous leakage and longer ICU stay in patients undergoing transthrocotomy than patients without transthrocotomy. However, regarding these respects, the included analyzable trials were too few (only 1 for each). It has been suggested that anastomosis at different locations may be associated with different incidences of anastomotic leakage [43]. However, our results have showed there was no significant difference for incidences of anastomotic leakage between transthoracic anastomosis and non-transthoracic anastomosis. Other analyses failed to show any significant differences on the incidences of complications, such as wound infection, cardiovascular complications, and hoarseness, between patients treated with transthrocotomy and those without transthrocotomy. These results were not supported by study of Boshier PR *et al* [39], which demonstrated that transthoracic group had significant higher incidences of respiratory complications, wound infections, and early postoperative mortality, whereas anastomotic leak and recurrent laryngeal nerve palsy rate were significantly more in the transhiatal group.

At the same time, with respect to the operation related events, transthoracic resection had significant differences from non-transthoracic resection in length of hospital stay, and was apt to more blood loss and longer operation time despite of no statistical differences. The operation time may be expensed in repositioning and redraping the patient, and in opening and closing a thoracotomy [12], [33], which did not appear to have an adverse effect on outcome. However, the prolonged operating time might aggravate the burden of patients with limited cardiopulmonary reserve [12]. And the hospital stay may be associated with the potential increased respiratory complications as well as the increased ventilation time, ICU stay, and tracheotomy caused by the respiratory complications after transthoracic resection [14], [15], [39]. So, transthoracic resection should be applied cautiously for patients with impaired cardiopulmonary function in the practice.

Also, there are some limitations on this meta-analysis. First, the included researches for analyzing were limited. Second, some data was obtained through indirect methods, such as hazard ratio from the survival curve and mean from median.

One of the major limitations of the study is the relatively high level of heterogeneity of the data, especially in the pooled analysis of operation related events. This arised because the more extensive technique of thoracotomy was preferred in treatment of Siewert type 1 tumors because they tended to have more metastasis in the lymph nodes of the middle or upper mediastinum than do type 2 tumors [44], and also selected for tumors with esophageal invasion of greater than 4 cm since a safe proximal margin was easily obtained [9], [15], however was avoided for older or patients with impaired cardiopulmonary function, although the basline characteristics of patients in both groups of the included studies were comparable. In addition, the included studies reported similar long-term survival rates with actually different resection techniques of thoracotomy may cause the heterogeneity of the data. The majority of type I tumors were histologically intestinal tumor growth pattern, while type II and III tumors were more commonly undifferentiated tumors and had worse prognosis [45]. Furthermore, early tumors (pT1) and the pN0 category were significantly more common in patients with type I tumors than in those with type II or III tumors [45]. Compared with patients with type III tumors, pN0 and pM0 categories were more common in patients with type I and II tumors [45]. This resulted in higher R0 resection rates in patients with type I and II tumors than in patients with type III tumors. These might be another source of heterogeneity. In spite of the limitations of this meta-analysis, by developing a detailed protocol before initiating the study, performing a cautious search for published studies, using objective methods for study selection, data extraction and analysis, and performing the subgroup analyses and sensitivity analyses, we have minimized the probability of bias as far as possible.

In conclusion, there were no significant differences of survival rate, postoperative morbidity and mortality between transthoracic resection group and non-transthoracic resection group. Both surgical approaches are acceptable, and that one offers no clear advantage over the other. However, the results should be interpreted cautiously since the qualities of included studies were suboptimal.
